# Toward brain-computer interface based wheelchair control utilizing tactually-evoked event-related potentials

**DOI:** 10.1186/1743-0003-11-7

**Published:** 2014-01-16

**Authors:** Tobias Kaufmann, Andreas Herweg, Andrea Kübler

**Affiliations:** 1 Department of Psychology I, University of Würzburg, Marcusstr 9-11, Würzburg 97070, Germany

**Keywords:** Brain-computer interface, Event-related potentials, P300, Tactile, Wheelchair, Dynamic stopping

## Abstract

**Background:**

People with severe disabilities, e.g. due to neurodegenerative disease, depend on technology that allows for accurate wheelchair control. For those who cannot operate a wheelchair with a joystick, brain-computer interfaces (BCI) may offer a valuable option. Technology depending on visual or auditory input may not be feasible as these modalities are dedicated to processing of environmental stimuli (e.g. recognition of obstacles, ambient noise). Herein we thus validated the feasibility of a BCI based on tactually-evoked event-related potentials (ERP) for wheelchair control. Furthermore, we investigated use of a dynamic stopping method to improve speed of the tactile BCI system.

**Methods:**

Positions of four tactile stimulators represented navigation directions (left thigh: move left; right thigh: move right; abdomen: move forward; lower neck: move backward) and N = 15 participants delivered navigation commands by focusing their attention on the desired tactile stimulus in an oddball-paradigm.

**Results:**

Participants navigated a virtual wheelchair through a building and eleven participants successfully completed the task of reaching 4 checkpoints in the building. The virtual wheelchair was equipped with simulated shared-control sensors (collision avoidance), yet these sensors were rarely needed.

**Conclusion:**

We conclude that most participants achieved tactile ERP-BCI control sufficient to reliably operate a wheelchair and dynamic stopping was of high value for tactile ERP classification. Finally, this paper discusses feasibility of tactile ERPs for BCI based wheelchair control.

## Background

Brain-computer interfaces (BCI) allow for direct communication between a person’s brain and technical devices without the need for motor control (for review, [1-4]). BCIs thus constitute a promising assistive technology device for people with severe motor impairment, e.g. due to neurodegenerative disease (e.g., [5-10]). Among many different applications, researchers suggested their use for wheelchair control (e.g., [11]), thus rendering BCIs of high value for people with severe paralysis who are not able to control a wheelchair by means of a joystick (e.g., [12]).

For example, people with intermediate spinal muscle atrophy (SMA, type II) are usually in need of a wheelchair at a young age. With progression of the disease, they may lose control of a wheelchair even by means of a small finger joystick. Control with eye-tracking devices is not feasible, as they obviously need the visual modality for observation of their environment during navigation. Facial muscles may also lose their reliability and are rapidly fatigued in frequent use [13]. With progression of disease, BCIs may become a feasible alternative for wheelchair control.

Among different input signals for BCI control, electroencephalography (EEG) appears viable for wheelchair control due to its high temporal resolution and portability. Most studies on wheelchair control by means of a BCI investigated sensorimotor rhythms (SMR) as input signal that can be modulated voluntarily by motor imagery (MI; [14,15]). It is possible to discriminate between different imageries or for example between imagery and rest. Each command is referred to as one class, e.g. left hand vs. right hand MI would be referred to as a two-class SMR-BCI paradigm. Different protocols have been suggested for wheelchair (or robot) navigation tasks that either analyze ongoing EEG activity (asynchronous control, i.e. a command can be delivered at any time; e.g., [11,12,16-19]) or analyze EEG activity at a given time window (synchronous control, i.e. a command can be delivered only at a certain time; e.g., [20-22]). The latter require cues that trigger the time windows and display them to the user. Such cues can be presented visually. However, to achieve SMR modulations without occupying the visual channel (i.e. visual cue on a screen), auditory-cued paradigms have been validated (auditory: e.g., [21,23]; auditory + visual: e.g., [20]). Furthermore, feedback can be presented through tactile stimulation units (e.g., [24,25]).

As any error made while controlling a wheelchair may immediately cause damage (or even danger for the patient), wheelchairs may be equipped with shared control systems, i.e. sensors that for example prevent collisions or regulate speed while approaching an object (e.g., [12,16,26-28]). Such shared control systems usually also dedicate parts of the movement control to the wheelchair as BCIs are not yet capable to operate on a full control level as possible with motor control [29]. One reason is, that the number of classes in SMR based BCIs is limited, as discrimination between different MI patterns becomes more difficult with increasing class number, and intensive training may be required [30]. Thus, researchers introduced paradigms that extrapolate different navigation commands from few MI classes only, e.g. translate three MI classes into six different commands [11] or two MI classes into three different commands [20,30].

Such translation, however, may require tasks that are more complex and entail slower rates for communicating commands. Furthermore, a general issue with motor imagery based BCIs is that for many participants SMR-BCIs are inefficacious or display large performance variations across runs [31-35]. However, reliability of BCI commands is particularly necessary for accurate wheelchair control. In a recent evaluation study, severely motor impaired end-users rated reliability of BCI applications controlled by event-related potentials (ERP) high [10]. ERP-based systems may thus constitute a more reliable alternative to SMR as input signal for wheelchair control, although users cannot actively modulate ERPs for control command generation but need external stimulation. ERP-BCIs make use of a so-called oddball-paradigm, i.e. rare but relevant stimuli are presented within frequent, but irrelevant stimuli. Users focus their attention by counting the rare target stimuli whilst ignoring all other (non-target) stimuli. Target stimuli will evoke more pronounced negative and positive potential fluctuations in the event-related EEG than non-target stimuli (for review on the paradigm, [36]). The most prominent potential in ERP-BCI systems usually is the P300, a positive deflection around 300 milliseconds post-stimulus ([37], its amplitude, shape and latency strongly varies with paradigms and subject-specific conditions; for review, e.g., [38]), which is why ERP-BCIs were often referred to as P300-BCIs (originally by [39]; for comparison of ERPs contributing to ERP-BCI performance [40]; for recent review [36,41-43]). By detecting the elicited ERPs, classification algorithms can identify the intended target selection and translate it into a control command.

Several ERP-based BCI systems for wheelchair (or robot) control have been proposed that differ strongly concerning the amount of control that is left to the user. Rebsamen and colleagues [44] proposed a system, which allowed users to select the targeted destination in a building (e.g. the kitchen) from a visually displayed ERP-BCI matrix. The wheelchair will then autonomously drive to the selected location. This fully transfers navigation control to the smart wheelchair and users can only interfere through selecting a stop mechanism that will terminate the movement. A similar level of control was proposed for control of a humanoid robot [45]. Users selected targeted objects or locations from a series of camera screenshots used as stimuli in an oddball-paradigm. The robot then autonomously approached and picked up the object. The advantage of such systems with which users select high-level goals (e.g. a location) while the system performs all low-level operations (steering toward the location) usually lies in its speed and accuracy. However, its performance fully depends on which and how many environmental conditions the device can handle. In addition, users may well prefer to have more process control on their side, as situational goals may change and the goal selection options of the smart wheelchair may not cover all goals.

An ERP-BCI for actual navigation control can easily be implemented by displaying direction arrows in a visual ERP-BCI matrix, i.e. the wheelchair is steered step by step by selecting the upcoming movement direction from a separately displayed matrix [46]. Iturrate and colleagues proposed a more advanced ERP-BCI for navigation control [47]. The authors equipped a wheelchair with a screen that displayed a reconstruction of the real environmental scenario in real time. Target locations were displayed in the reconstruction model and could be selected using an ERP-BCI. Consequently, the system leaves more decisions to the user, yet the actual target locations are computed by the smart wheelchair, i.e. users can only select those target locations that are recognized as possible locations by the detection sensors. This system was recently developed further for control of a telepresence mobile robot [48]. Furthermore, different input signals can be combined for wheelchair control in a hybrid approach (e.g., [49]). Long and colleagues [49] implemented a system that controlled direction by means of SMR modulation and speed with a visual ERP-BCI.

Although visually elicited ERPs usually provide best classification accuracies [50] and thus highest information transfer rates compared to other modalities (for review, e.g., [36]), there are several issues with regard to wheelchair control. The same issues apply to BCIs based on steady-state visual evoked potentials (SSVEP, e.g., [51]) (1) Visual stimulation requires a display mounted in the visual field of the user, which is critical for those with severe impairment not able to move the neck for looking past the screen to observe their environment they navigate through. (2) Users cannot observe their environment in the process of target selection, as they need to pay attention to the visual stimulation. (3) Changing light settings may negatively influence the efficacy of BCIs that rely on visual stimulation (e.g. due to bright sun).

In light of these restrictions, Brower and van Erp proposed to tactually elicit ERPs for BCI control [52]. Such tactile BCIs use tactile vibration units (called tactors) placed on participants’ body, e.g. on hands and wrists [50], on different positions around the waist [52-54] or on the back of participants [54]. Similar to the visual oddball-paradigm, tactors are stimulated randomly (i.e. they vibrate for a short time) and participants focus their attention on one of the tactors (target) whilst ignoring all others (non-targets). Stimuli will elicit distinct ERPs among which the most prominent is the above described P300 component ([54]; for a thorough investigation of tactually-evoked ERPs in a BCI setting). Brouwer and van Erp [52] investigated how stimulus uncertainty (i.e. the number of stimuli used) and stimulus timing affect classification accuracy and found equal accuracies for two, four and six tactors. For stimulus timing, they found similar parameters feasible as used for visual ERP-BCIs. Thurlings and colleagues [54] found, that placement of tactors significantly affected offline BCI performance in a paradigm that applied tactors for control-display mapping (i.e. mapping between navigation directions and tactor location). A placement that was congruent with the navigation environment provided best results. Recently, a case study reported tactile stimulation feasible for reliable elicitation of ERPs in a patient with classic locked-in syndrome [55]. Results were more robust in the tactile than in the auditory or the visual domain. Our current study is based on these results that established a basis for tactile ERP-BCI based navigation.

In contrast to the above described studies on wheelchair control that use SMRs, SSVEPs or visually-evoked ERPs as input signal, this study investigated feasibility of tactually-evoked ERPs for wheelchair control. (1) We exposed participants to a virtual environment. Participants steered a virtual wheelchair in real time by selecting one of four tactor locations. This approach allowed us to investigate how more complex (and realistic) scenarios affect user performance. Navigation tasks can be regarded as more complex, as users individually decide on the path they take and as processing of their environment may distract them. (2) Recently, researchers reported great benefit of dynamic stopping methods for visual and auditory BCIs (e.g., [56-60]; for comparison of techniques [61,62]). The proposed algorithms stop the stimulation cycle when classification reached sufficient probability for identification of the intended target from the event-related EEG. Thus, they dynamically adjust the number of stimulation cycles based on users’ individual brain signals. In this work, we investigated the potential of dynamic stopping on performance and timing in tactile ERP-BCIs. (3) Finally, we evaluated device satisfaction following the user-centered approach [10,63].

## Methods

### Participants

N = 17 healthy participants were recruited for this study. We excluded one participant due to incompliance with the experimental protocol and one participant stopped before the end of the experiment. The final sample thus comprised N = 15 participants (12 female, mean age: M = 21.8 years, SD = 2.9, range 18–27 years). All had normal or corrected-to-normal vision and none reported any neurological disorders. All participants were naïve with regard to tactually evoked ERP-BCIs. We conducted the experiment in accordance with standard ethical guidelines as defined by the Declaration of Helsinki (World Medical Association) and the European Council’s Convention for the Protection of Human Rights and Dignity of the Human Being with regard to the Application of Biology and Medicine (Convention on Human Rights and Biomedicine). All participants gave written informed consent prior to the study. The study was approved by the ethics committee of the Institute of Psychology at University of Würzburg, Germany.

### Equipment and data acquisition

Eight tactile stimulators, i.e. vibrate transducers (C2 tactors; *Engineering Acoustic Inc., Casselberry, USA*), were grouped into pairs of two and attached to a participant’s left thigh (top, toward knee), right thigh (top, toward knee), abdomen (above navel) and lower neck (at the height of C4 to C8) using Velcro® belts. Prior to the experiment participants had the opportunity to stimulate all tactors individually, to ensure that they adequately perceived all stimulations. During the experiment, each pair of tactile stimulators constituted one target, i.e. two tactors at close position were stimulated simultaneously. We found that grouping two tactors into one target facilitated participants' recognition of stimuli in a pilot study. Stimulus duration was set to 220 ms and inter-stimulus interval to 400 ms. Stimulation frequency was 250 Hz.

EEG was acquired from 16 passive Ag/AgCl electrodes at positions Fz, FC1, FC2, C3, Cz, C4, CP1, CP2, P7, P3, Pz, P4, P8, O1, Oz and O2 ([5]) with ground and reference being applied to right and left mastoid respectively. Impedance was kept below 5 kΩ. Signals were amplified using a g.USBamp (g.tec Engineering GmbH, Graz, Austria) and recorded at a sampling rate of 512 Hz. Band pass filtering between 0.1 and 60 Hz and notch filtering between 48 and 52 Hz were applied online.

### Software implementations

#### Tactile stimulation

We implemented control of the C2 tactor API in C++ and integrated it into the BCI2000 software (Version 3.0; [64]). We modulated the P3Speller module, usually used for communication of characters (for details on the procedure see [39]), such that flashing of the visual character matrix triggered stimulation of tactor pairs (see section “Equipment and data acquisition”). In a 4×4 character matrix, flashing of row 1 or column 1 would trigger stimulation of tactor pair 1, row 2 or column 2 would trigger tactor pair 2, etc. Consequently, a 4×4 matrix triggers four possible targets (the diagonal). The underlying spelling matrix was invisible to the participants.

#### Feedback paradigms

**Figure 1:**
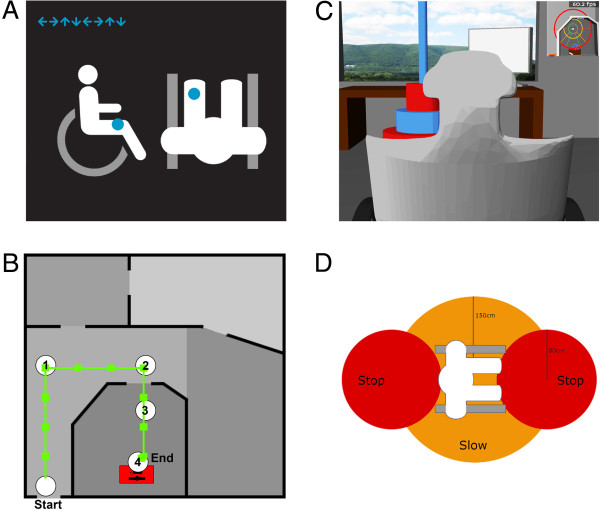
**Experimental design. (A)** Screenshot of the display presented during the calibration phase. The current target tactor was presented schematically in top and side view. The arrows on the top left indicate the consecutive targets of the run. **(B)** Top view of the floor plan. Four checkpoints were inserted into the building and participants had to target one after another until reaching a desk at checkpoint 4. **(C)** Screenshot of the virtual environment (view from behind the neck support of the wheelchair). The screenshot was taken shortly before reaching the final checkpoint (blue/red stack) close to the desk (left center of the screenshot). In the upper right corner, position tracking was provided for orientation in the building. **(D)** Collision zones of the wheelchair. When frontally approaching an object (i.e. an object enters the “stop” zone marked in orange), the wheelchair would stop to prevent collision. Furthermore, it would slow down when any objects entered the “slow” zone (green ellipse around the wheelchair).

Participants were guided through the calibration and copy task runs (see section “Study design”) such that the current target was displayed on a screen, i.e. target positions on the body were presented in a schematic side- and top view. Figure 1A provides a screenshot of the presented display during the calibration phase. The same display was also presented during the copy task runs except that feedback on the outcome of classification was provided in real time. We implemented the paradigms in Python 2.5 (using Pygame 1.9 and PyOpenGL 3.0) and connected them to BCI2000 via user datagram protocol (UDP). Feedback paradigm and BCI2000 were executed on separate computers.

#### Virtual environment

We created a 3D-model of a virtual building in Blender 2.6 (Blender Foundation, Amsterdam, Netherlands). It comprised a single floor with four rooms and a corridor. Figure 1B displays a top view of the floor plan. We also modeled a wheelchair and several objects (table, checkpoint flags) in Blender and generated corresponding textures with Gimp 2.8 (http://www.gimp.org, GNU Image Manipulation program). The Panda3d game engine (Version 1.7; Entertainment Technology Center, Pittsburgh, USA) was used to accomplish motion of the wheelchair through the building. Finally, the virtual environment was connected to BCI2000 via UDP. Figure 1C provides a screenshot of the virtual environment. Participants controlled the wheelchair from a third person perspective (view from behind the neck support of the wheelchair). We chose this perspective as from a first person perspective the wheelchair would not have been visible and participants could not have looked around as would be possible in a real wheelchair setting or virtual environment. As the scenario displayed on the screen was restricted to one view, we consequently chose a view from which they could perceive the wheelchair and their environment. In the upper right corner, a top view map provided position tracking to support orientation in the building.

The virtual wheelchair was equipped with collision sensors imitating the behavior of an intelligent wheelchair. The collision system was implemented independent from the one incorporated in Panda3d’s game API, as this pre-set collision system allows for sliding along walls. This would not be feasible for wheelchair control. The wheelchair was thus equipped with collision sensors that would either stop the wheelchair (prevent collision with an object and/or sliding along it) or slow down the wheelchair’s speed to enable for more accurate control (e.g. when passing through a door). Figure 1D illustrates the collision zones of the wheelchair. Detection of objects within the forward or backward collision zones immediately stopped all movement in the specific direction and the wheelchair ignored all further commands in this direction until the zone was cleared again. By utilizing generous forward and backward collision zones we ensured that collision free turning is possible after the wheelchair stopped. Detection of objects within the “slow mode” collision zone reduced the movement and turning speed down to 50% of the original value until the zone was cleared again.

Each time a pair of tactors was classified as target (left, right, forward or backward; section “Equipment and data acquisition”) the wheelchair would either move by 1 virtual meter into the desired direction or turn to the requested side by 45 degrees.

We placed four checkpoints in the building. They illustrated the task of moving along a corridor through a door into the office room to approach the desk. The optimal path to fulfill this task comprised 16 commands with no more than 5 commands in between two check-points (see Figure 1B).

#### Offline and online classification: dynamic stopping and static stopping

We refer to classification based on data acquired during a calibration run as offline classification, whereas online classification is classification that is performed during ongoing data collection and results in immediate feedback to the user.

During online runs, data were streamed into MATLAB 2010b (The Mathworks Inc., Massachusetts, USA) using Fieldtrip ([65]; http://fieldtrip.fcdonders.nl). Online classification (stepwise linear discriminant analysis, SWLDA, 800 ms post-stimulus; as e.g. used in [39,66,67]) was then performed in MATLAB and results communicated to the feedback applications by means of UDP.

**Figure 2:**
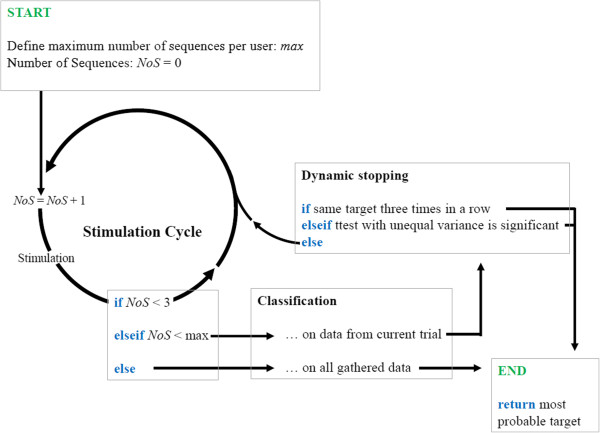
Decision flow chart of the dynamic stopping method.

We implemented a dynamic stopping based on a combination and modification of two recently published dynamic stopping methods ([56,57], see introduction). Figure 2 illustrates the decision tree. The tree comprised three basic rules as follows. (1) A minimum number of three sequences were collected for classification. (2) If no decision could be made after gathering a predefined maximum number of sequences (NoS), the most likely target was classified from all gathered sequences of the trial. The maximum number of trials was adjusted for each participant separately based on results from calibration (minimum NoS to reach offline performance estimation of stable 100% plus two sequences; described in detail in [68]). (3) A dynamic stop could be performed if the most likely target was the same three sequences in a row (modified from [57]) or if a *t*-test with unequal variance performed on so far gathered samples was significant at an alpha level below 10% (modified from [56]). The alpha level was chosen after pilot testing.

We compared dynamic stopping to the commonly used static stopping, i.e. each trial comprised a fixed number of sequences that were all used for classification. The number of sequences was equal to the maximum number of sequences used in the dynamic stopping run.

### Study design

Before the experiment, participants were instructed and tactors were placed (see section “Equipment and data acquisition”). Participants had the possibility to adjust tactor positions by a few centimeters until they perceived all stimulations equally well. To familiarize the participants with the floor map and with the control principle of the virtual wheelchair, they used a keyboard to move the wheelchair through the virtual environment during EEG preparation.

The actual experiment consisted of one calibration run (predefined task; data is used to compute classifier weights), two copy tasks (predefined task; used to evaluate classifier performance online) and finally the main goal of the study, i.e. one task aiming at navigation through the virtual building. Duration of calibration was 10 min. Duration of copy and navigation tasks were participant specific depending on their performance (see section “Results”). One calibration trial comprised 15 stimulation sequences per tactor pair, i.e. each tactor pair vibrated 30 times (one sequence corresponding to four row and four column flashes in the visual matrix; see section “Software implementations - Tactile stimulation”). Calibration was performed with eight trials (each tactor pair was twice the target). If offline analysis revealed a performance below 100% after these eight trials (when including all sequences into classification), we repeated calibration once. After calibration, participants performed two copy task runs. One copy task run included static number of sequences, i.e. each trial comprised a maximum number of sequences before classification. A second copy task run introduced the above-described dynamic stopping method. This allowed for with-in comparison of performance achieved with and without dynamic stopping. During both copy tasks, immediate feedback on classification outcome was provided to the participants. As for the calibration run, each tactor pair was twice the target, resulting in eight trials per copy task run. Participants then moved on to control of a virtual wheelchair and tried to navigate along the predefined route (see section “Software implementations – Virtual environment”). When reaching one of the four checkpoints, they took a break of approximately one minute before moving on (the BCI was manually switched off during this time by the experimenter). The number of trials during navigation varied dependent on the participants’ performance. In the optimal path (Figure 1B) selection of the “move forward” command was required most frequently. However, as errors had to be corrected, the number of required commands per navigation direction differed between participants.

### Offline data processing of ERPs

EEG data were filtered between 0.1 and 30 Hz (FIR equiripple) and divided into segments of 800 ms post-stimulus. Determination between targets and non-targets was quantified by computing R^2^ values. For computing the grand average of R^2^ values we Z-transformed (Fisher’s Z) the square root of the determination values for each participant and electrode, averaged across participants and finally retransformed and squared these grand averages.

### Analysis of system performance

In the virtual environment, performance estimation is difficult, as different paths may be feasible for reaching the checkpoints. For example, after an error participants may either steer back by one step or take a different path to approach the next checkpoint. Thus, we asked participants to report during the breaks whether or not the selected targets were the desired targets and performance was computed based on their reports. To control for false reporting, we manually went through each decision and decided if it was goal-oriented. Finally, we aligned these two analyses. Except for two selections, these decisions were similar to the subjects reports (265 selections in total; from the two selections one would slightly increase performance estimate, one would slightly decrease performance estimate). Therefore, we consider adequate to estimate performance based on subjects reports.

The impact of shared control was determined from the number of collisions and the number of times when sensors for slowing down speed were active. Furthermore, we computed the time required for delivering commands from the duration of stimulus and inter-stimulus intervals. Classification time, wheelchair movement duration and duration of the breaks the participants took at each checkpoint were not taken into account. Thus, the reported time is system independent and includes only the mandatory time needed for stimulation.

Furthermore, following the user-centered approach we validated the system based on user reports. Participants rated their confidence with tactile ERP-BCI based wheelchair control with forced choice questionnaires. The questions covered learnability, strain, level of control, speed of the system and participants’ trust in the used BCI technology [10].

### Statistical analysis

We checked data of achieved BCI performance for normal distribution using Lilliefors - Kolmogorov Smirnov tests. Due to non-normal distributions, we performed pairwise testing with the Mann–Whitney *U* test. Bonferroni correction to 5% alpha levels is indicated. Statistical analysis was performed in Matlab 2010b.

## Results

**Figure 3:**
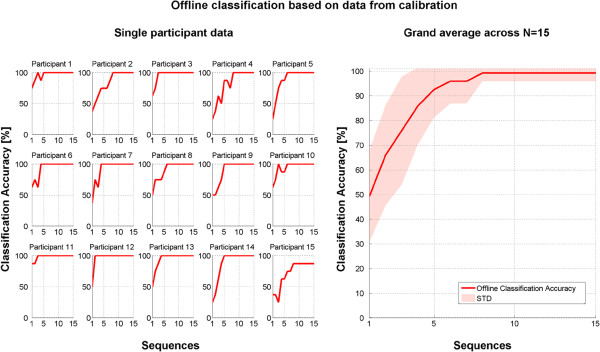
Offline classification accuracy estimated from calibration data for each individual subject (left) and averaged across all subjects (right).

Five participants repeated calibration once due to insufficient offline performance estimates after the first calibration run. Figure 3 displays offline classification performance: N = 14 of 15 participants achieved offline classification accuracy of 100%. Their average number of sequences required to reach Stable 100% offline accuracy (i.e. retaining 100% performance when adding further sequences) was M = 4.9 (N = 14, SD = 1.8, range: 2–8). This would correspond to an average time of M = 24.3 s per command. Offline performance for participant 15 was estimated Stable 87.5% with eight sequences, but did not further improve when calibrating on all sequences.

### Dynamic vs. static stopping

**Figure 4:**
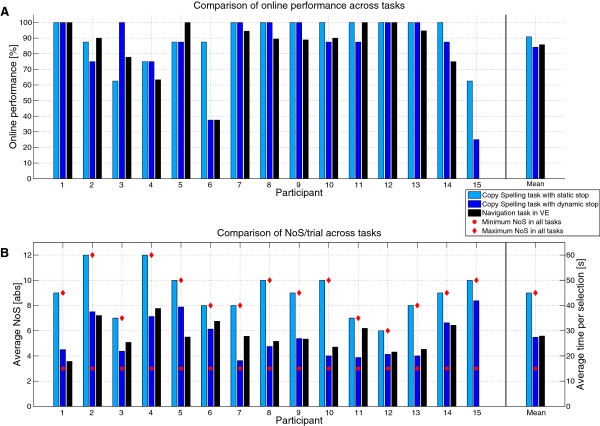
**Comparison of task performance and duration in all three online tasks for each participant. (A)** Online performance. **(B)** Number of sequences and required time per selection.

We validated tactile stimulation for ERP elicitation online in two copy tasks. Participants gained overall high accuracy levels in both tasks (see Figure 4A). Average accuracy with static number of sequences was M = 90.8% (SD = 13.7, range 62-100%) and nine of 15 participants performed without errors. The time needed to fulfill the task with static stopping ranged from 4.2 to 8.2 min (M = 6.1, SD = 1.2 min), whereas the time needed to fulfill the task with dynamic stopping ranged from 2.6 to 5.4 min (M = 3.7, SD = 1.0). Performance did not significantly decrease when introducing dynamic stopping (N = 15, Z = 0.70, p = .48; M = 84.2%, SD = 23.4), i.e. most participants maintained the performance level achieved with static number of sequences. However, performance for two participants (participant 6 and 15) severely decreased - for participant 15 even to chance level (25%). Furthermore, we investigated if errors were equally distributed across targets. The total amount of errors did not differ between the targets (left: 10% errors of all left target selections; right: 11.7%; forward: 13.3%; back: 15%; N = 15, H (3) = 0.97, p = .81, Bonferroni adjusted alpha level: α = .0083).

Figure 4B depicts the average number of sequences needed to deliver a command. In line with previous reports, the number of sequences significantly decreased in the dynamic stopping copy task (N = 15, Z = 3.81, p < .001). Consequently participants on average needed M = 27.2 seconds per selection as compared to M = 44.6 seconds in the task with static number of sequences.

### Wheelchair navigation

**Figure 5:**
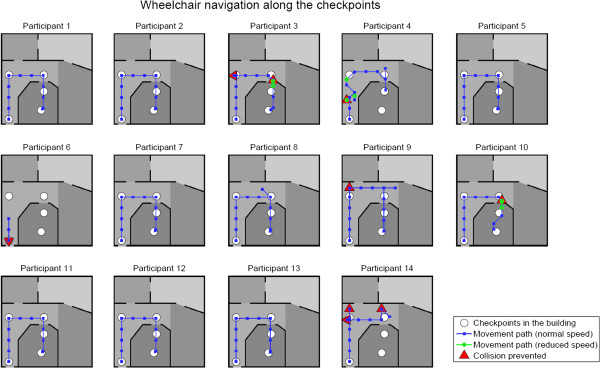
Path along which participants steered the virtual wheelchair.

Participant 15 did not perform the navigation task as the performance decreased to chance level when using dynamic stopping in the copy task (section “Results – Dynamic vs. static stopping”). Thus, only N = 14 of 15 participants performed the navigation task through the virtual building. For each participant, Figure 5 illustrates the path along which they steered the virtual wheelchair. Importantly, N = 11 participants reached the targeted desk at checkpoint 4 and four participants made no error. Although the navigation task can be regarded as more complex than a simple copy task, performance did not significantly decrease in the virtual environment (N = 14, Z = 0.33, p = .74). Average accuracy was M = 85.8% (SD = 17.6, range 37.5-100%) with a mean of M = 5.58 sequences. Three participants, however, could not successfully finish the task and performed the experiment only until they communicated to prefer canceling. Two of them at least managed to pass the corridor before quitting whereas participant 6 again had almost no control (due to dynamic stopping, see section “Results – Dynamic vs. static stopping”) and thus canceled the experiment early.

In contrast to the copy tasks that involved no correction of errors, wrong selections in the virtual environment had a direct impact for the further navigation task, i.e. errors had to be corrected. Alike intelligent wheelchairs proposed in robotics research, the virtual wheelchair was thus equipped with simulated shared control sensors. Most participants (N = 8) did not navigate into any situation where these sensors were needed. Collision was prevented once for N = 4 participants, twice for participant 3 and five times for participant 14. Sensors for slowing down speed of the wheelchair were active for two participants when passing the door to the office room. Hence, they managed to enter the room and reach the checkpoint. For participant 4 these sensors were activated three times when passing close to a wall, but did not have an effect on the navigation, i.e. they were instantly turned off again with the next movement of the wheelchair (see Figure 5). Table 1 summarizes participants’ individual navigation task performances and task durations.

**Table 1:** Summary of participants’ individual performances in the wheelchair navigation task

**Participant**	**Final checkpoint reached**	**Time needed [min] (b.c. = before canceling)**	**Accuracy (sensitivity) [%]**	**Specificity [%]**	**Average time needed per selection [s]**	**Average number of sequences per selection [abs]**	**Collision sensors needed [abs]**	**Sensors for slowing the wheelchair needed [abs]**
1	x	8.8	100.0	100.0	17.7	3.6	-	-
2	x	20.8	90.0	96.7	35.7	7.2	-	-
3	x	21.0	77.8	92.6	25.2	5.1	2	1
4	-	36.0 b.c.	63.3	88.5	38.5	7.8	1	3
5	x	12.8	100.0	100.0	27.3	5.5	-	-
6	-	7.8 b.c.	37.5	79.2	33.5	6.8	1	-
7	x	15.0	94.4	98.2	27.6	5.6	-	-
8	x	14.4	89.5	96.5	25.6	5.2	-	-
9	x	14.3	88.9	96.3	26.6	5.3	1	-
10	x	14.9	90.0	96.7	23.3	4.7	1	1
11	x	14.3	100.0	100.0	30.7	6.2	-	-
12	x	10.3	100.0	100.0	21.4	4.3	-	-
13	x	12.7	94.7	98.2	22.5	4.5	-	-
14	-	22.7 b.c.	75.0	92.8	31.9	6.4	5	-
	*Total:* N = 11	*Mean: 14.5 (excl. those who canceled)*	*Mean:* 85.8	*Mean: 95.4*	*Mean:* 27.7	*Mean:* 5.6	*Total:* 11 (N = 6)	*Total:* 5 (N = 3)

### ERP differences in target vs. non-target trials

**Figure 6:**
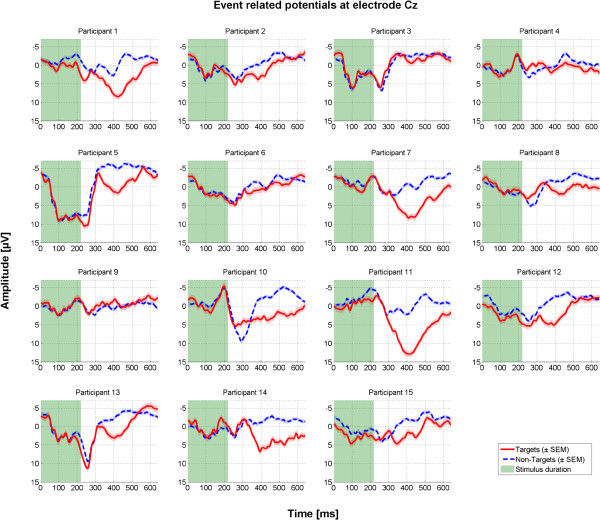
Average event-related potential at electrode Cz for all N = 15 participants based on calibration data.

**Figure 7:**
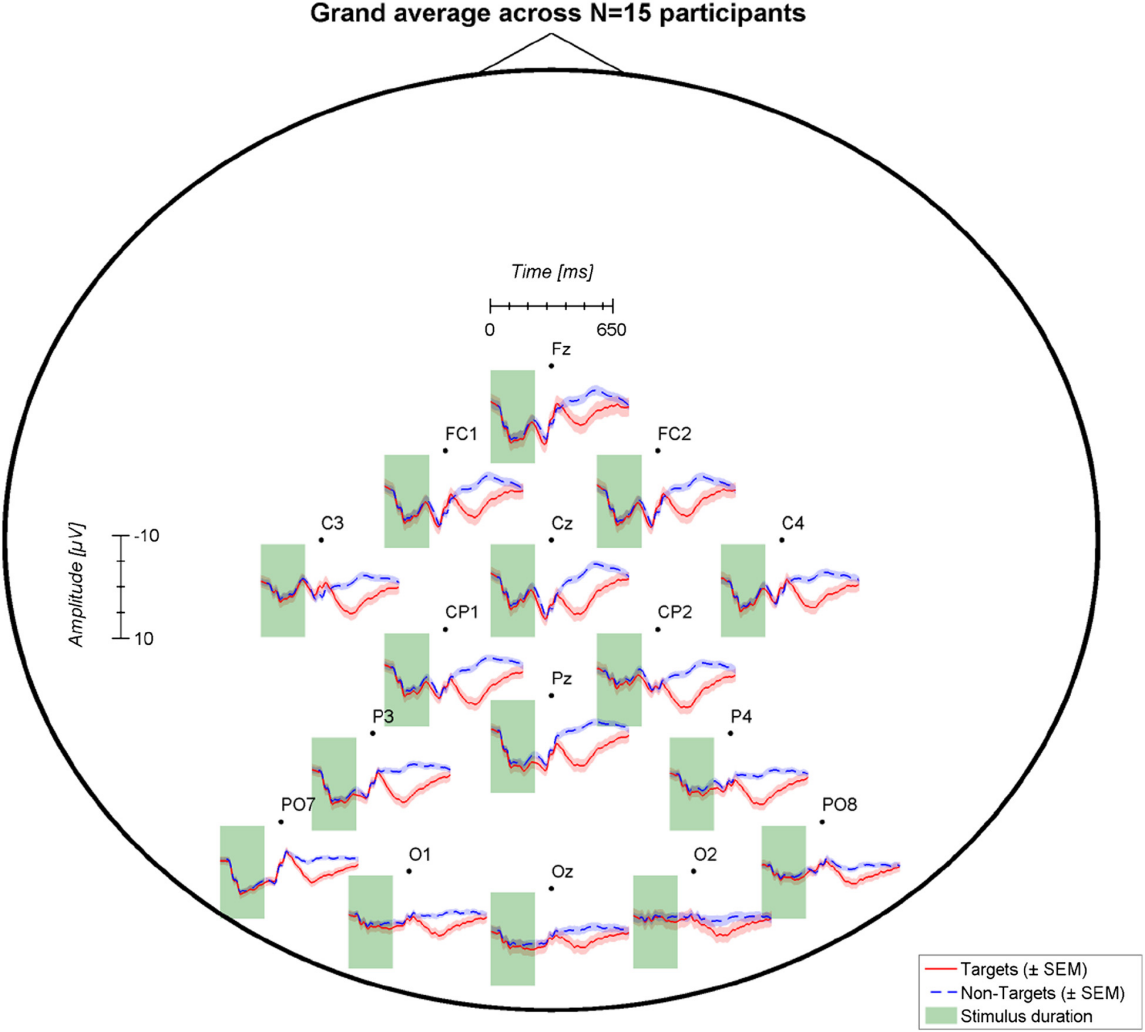
Topographical representation of the grand average event-related potential across N = 15 participants based on calibration data.

During stimulus duration, tactile stimulation of non-target positions also evokes an event-related response as participants directly perceive all stimuli on the body and cannot easily ignore them. Yet after around 300 ms, target and non-target signals diverge. Target stimulation elicits a P300, whereas non-target stimuli often entail a negative ERP in the period between 300 and 500 ms post-stimulus. ERP responses differed considerably between participants, yet for all of them discrimination between target and non-target stimuli was possible (see Figure 6). Figure 7 provides a topographical map of the grand-averaged ERPs across all participants based on calibration data.

**Figure 8:**
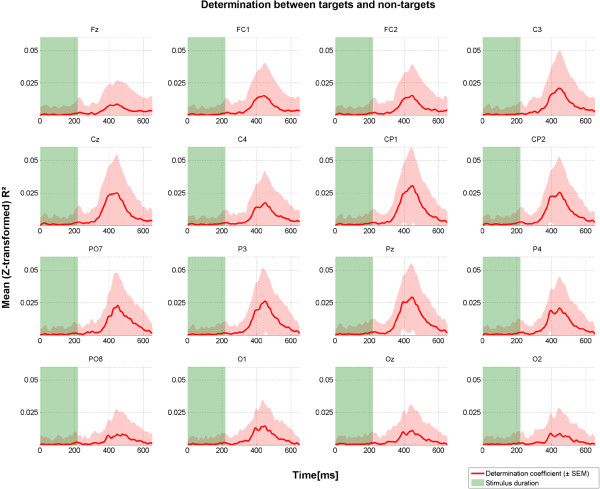
**Grand average across N = 15 participants of determination coefficients over time for all electrode sites.** Values were Fisher-Z transformed before averaging. Results are based on calibration data.

We further computed the determination coefficients to investigate which features contribute most to classification. As depicted in Figure 8, the centro-parietal electrodes contributed most to discrimination between targets and non-targets. Determination coefficients were highest between 400 and 500 ms, i.e. in the time window of the tactile P300.

### Subjective validation with questionnaires

We further explored system performance using forced choice questionnaires with the four choices “I do not agree at all”, “I do not really agree”, “I mostly agree”, “I fully agree”. Table 2 depicts the results. All participants were confident with learning how to control the wheelchair and – except participants 6 and 14 – with reliability of control. As expected, responses to questions on learnability and reliability depended on participants’ task performance. With regard to strain and speed participants’ answers were independent of their actual performance (Kendalls Tau τ = .06, p = .86). For example, participant 5 who did not perform any error in the virtual environment stated that control was too demanding.

**Table 2:** Questionnaires on satisfaction with the tactile ERP-BCI based wheelchair control

**Question**	**I do not agree at all [%]**	**I do not really agree [%]**	**I mostly agree [%]**	**I fully agree [%]**
Control of the wheelchair was quickly learnable	0.0	0.0	71.4	28.6
The wheelchair correctly recognized the delivered commands	0.0	14.3	71.4	14.3
I always had full control over the wheelchair	14.3	0.0	71.4	14.3
Control of the wheelchair was too demanding	50.0	21.4	28.6	0.0
Control of the wheelchair was too slow	0.0	64.3	35.7	0.0

## Discussion

### Tactile ERPs for BCI based wheelchair control

We exposed participants to a virtual environment and asked them to navigate a virtual wheelchair by means of a tactually evoked event-related potential based BCI. Our results are promising in that most of the participants reached the final checkpoint and that only few participants needed shared control.

Importantly, N = 14 of 15 participants reached 100% offline classification accuracy and one further participant had an offline accuracy level of 87.5%. In all three online tasks, performance of N = 11 participants remained above 70%. For two further participants performance may have remained high (participant 6) or at least medium (participant 15) if we would not have switched to the dynamic stopping method. Tactile ERP-BCIs may thus offer a valuable alternative to motor imagery based BCIs considering the findings that many SMR-BCI users do not gain sufficiently reliable SMR control [31-35]. Also, SMR-BCIs usually require a longer calibration phase than ERP-BCIs and intensive user training may be necessary to achieve a good level of control, specifically in people with neurodegenerative disease [8]. However, performance varied considerably between participants implying the need for testing larger groups for generalization of results, which is hardly ever the case in studies that use BCI for wheelchair control (e.g., N = 2 in [11,12,16,30,46]; N = 3 in [69]; N = 5 in [20,44,47,48]; and N = 6 in [22]). Furthermore, often healthy users with prior BCI experience were selected thereby also hampering generalization of results (e.g., [30,70]). Since all our participants were naïve with regard to tactile ERP-BCIs, we speculate that a studious learning of tactile perception (in particular learning to ignore irrelevant tactile stimulations) may further enhance their performance. Furthermore, rebuilding classifiers based on more data input may increase performance, as the short calibration performed at the beginning of the experiment may not be sufficient.

Consequently, in case more data would further enhance classifier accuracy, generic models could be of high value to shorten calibration time (i.e. building a classifier based on data from a large pool of participants; e.g., [71,72]). Also, such models may increase performance of those participants who do not achieve accurate control with their individual classifier [73]. However, our results show large inter-individual differences of the ERPs elicited post-stimulus. In line with previous reports (e.g., [54]) the tactually-evoked P300 peaked at central electrodes with an average latency around 400-500 ms. Centro-parietal electrodes contributed most to classification accuracy. Considering the varying ERP responses across participants, recording from more electrode sites could further enhance subject-specific ERP detection and facilitate investigation of generic models.

Our study design built on prior work on tactile ERP elicitation. Brouwer and van Erp [52] found no performance difference with regard to a number of two, four or six tactile stimulators. We thus implemented a system based on four tactors representing direction control units. Thurlings and colleagues [54] investigated how congruent tactor positioning affects task performance. They positioned a monitor vertically or horizontally in front of participants. A control display mapping was realized with tactors positioned either congruent with monitor angle (i.e. horizontal tactor positions around the waist for horizontal monitor placement and vertical tactor positions on the participants’ back in the case of vertical monitor placement) or incongruent (i.e. horizontal tactor positions around the waist and vertical monitor placement). The authors demonstrated that a congruent setup yielded increased P300 amplitudes and thus increased estimated BCI performance. Therefore, in our study we aligned tactor placement with movement directions. With regards to stimulus timing we opted for an on-time of 220 ms and an off-time of 400 ms, i.e. a similar timing than the baseline condition from Brouwer and van Erp [52] in experiments 1 and 2 (188 ms on-time, 367 ms off-time). The authors suggested matching on- and off-times and found this condition to enhance bit-rate while maintaining the performance level. Such adjustment may thus also be feasible for our proposed system. However, due to the increased probability of ERP overlap when reducing off-times, we chose the longer duration.

In contrast to Brouwer and colleagues [52], who chose only the front tactor as target, our calibration and online copy tasks comprised equally often all tactors as target. Our results thus account for perception differences or attention difficulties between different body locations. Some participants may for example perceive the front target (close to the navel) stronger than the back target. In our study participants performed equally well on selection of tactors, i.e. in total participants did not perform significantly more errors on any of the targets than on others. Especially in light of a BCI with manifold selection options (realized placing many tactors on the body), it is inherently important to adjust tactor locations according to users’ reports so that they perceive all targets (approximately) equally well.

In line with previous reports from visual and auditory ERP-BCIs (e.g., [56-60]; for comparison of techniques [62]), dynamic stopping was of high value also for tactile ERP-BCIs. Participants greatly benefited in terms of time needed to deliver commands, thereby increasing speed of the system. Importantly, the reduced number of sequences in the dynamic stopping copy task did not affect performance (no significant difference between static and dynamic stopping copy task performance) except for two participants who displayed a strong performance drop during dynamic stopping. Hence, these participants did not benefit from dynamic stopping. From the offline classification results as well as from task performance in the copy task with static stopping we assume that participant 6 may have successfully performed the navigation task when using a static number of sequences. As participant 15 did not perform a navigation task, we do not know whether the drop in performance was due to bad performance in one run or due to dynamic stopping. In a comparison of dynamic stopping methods, Schreuder and colleagues [61] reported that some methods decrease performance of participants with less discriminative data. Considering the fact that offline classification performance of participant 15 displayed aggravated discriminability compared to other participants’ data, the performance drop may be attributed to dynamic stopping. For all participants, user specific parameter adjustment (as performed by e.g., [56]) could have further increased performance of the dynamic stopping method, especially in the case of those two participants. This may have prevented the algorithm from stopping too early although classification of the target was not sufficient.

Validation of the system based on questionnaires revealed that tactile ERP-BCI based wheelchair control is quickly learnable by naïve participants. Device satisfaction regarding reliability and control was mostly positive. However, evaluation results for demand of control and speed of the system varied and were independent of users’ performances. To better estimate these aspects, longer navigation tasks will be needed. On the one hand, learning to perceive stimuli may positively affect the demands for the user, on the other hand long navigation tasks may further increase demands on attention. Users of such systems in daily life navigation tasks may judge speed of the system more critically.

### Limitations and future experimentation

This study explored feasibility of the proposed BCI system in healthy users. We assessed user confidence with forced choice questionnaires to identify remaining issues and how they depend on task performance. However, validation may strongly vary with users’ health and with their actual dependence on the technology. Further research must investigate use of tactile ERP-BCIs by the actual target population. In the process of user-centered BCI development, potential end-users with severe motor impairment should be integrated into the design process at an early stage, so that research can specifically account for their needs and requirements ([6,10,63,74-76]). Furthermore, the effect of proposed improvements may well be larger in patients as compared to healthy participants (as recently found for a modification of visual ERP-BCIs; [7]). In particular, we suggest including patients with SMA type II who we consider a potential target group for use of BCI based wheelchair control. With progression of disease, they usually lose the ability to control a wheelchair with a joystick. Eye-tracking devices would occupy the visual channel needed for observation of their environment and devices based on facial muscles may be too fatiguing. Progression of the disease is usually slower than for example for patients with amyotrophic lateral sclerosis, which renders it more feasible to learn device control when needed. Cheliout-Heraut and colleagues [77] reported abnormalities of somatosensory-evoked potentials in a sample of SMA children (type I and II). Yet, these abnormalities occurred far less frequent in SMA type II than in SMA type I. As somatosensory-evoked potential abnormalities were more pronounced in the lower limbs, the proposed tactor positions may not be feasible and thus adjusted individually. The same issue may apply to other types of diseases or injuries, e.g. in the case of spinal cord injury tactile perception on the legs is usually lost. Thus, in all cases, the system requires individually-tailored adjustments based on the sensory perception capabilities of patients.

Generalization of results may be limited with regard to the complexity of the navigation task performed in this study. The path did not require users to select all direction options. From the results of the copy-task, however, it appears unlikely that more errors would have occurred for a different path. Yet, future testing of the system should be performed with several different tasks over a longer period of time. In addition, a vivid environment, in which users need to react to changing settings, could provide useful insights in feasibility of tactual ERP-BCI systems under such, more realistic conditions. Finally, generalization may be limited as the third person perspective and the position tracking used in this study may have positively influenced navigation ability, e.g. estimation of distances. However, in a virtual environment it may be more difficult to estimate distances than in a real world setting. Thus, the benefit of position tracking and perspective may be negligible as compared to the benefit of navigating in a real environment.

However, in its current state the system bears some major drawbacks. (1) Some users reported that focusing on tactile stimulation was too demanding in a long navigation task. Thus, stimulation should be enhanced so that users perceive stimuli better. Furthermore, training in several sessions could be conducted to decrease users' workload. Halder and colleagues recently demonstrated, that performance with an auditory ERP-BCI can be improved with training [78]. Zickler and colleagues [10] demonstrated for visual ERP-BCIs that subjective workload of a naïve, severely motor impaired, potential end-user could be strongly decreased the more sessions were conducted, i.e. in his first session he rated workload rather high (49 of 100 on a linear scale) but decreased his rating to 15 in the last session. (2) The average time to deliver a command was roughly 28 seconds, ranging from 17.8 to almost 38.8 seconds. For effective wheelchair control, speed should be further enhanced, e.g. by implementing other dynamic stopping techniques or by increasing the signal-to-noise ratio of the recorded ERPs [7,79]. As already addressed above, decreasing the off-time parameter of the system may also enhance speed. (3) The herein tested system is synchronous and not able to detect if a user wants to deliver a navigation command or perform any other task. For example, users may want to interrupt navigation and perform navigation-independent actions (e.g. communicating, reading, observing). It is thus inherently necessary to implement an asynchronous system that will account for such situations [80-82]. (4) Finally, we did not implement an option that rapidly allows for stopping the wheelchair. Once users delivered a movement command, they would hand over full control to the wheelchair, i.e. only its sensors could stop the wheelchair in case of an obstacle. Currently, if they delivered a wrong command, the wheelchair would still perform the action if the requested movement would not interfere with navigation barriers. Implementation of such correction method could be based on residual muscle activity or on other BCI signals in a hybrid approach (e.g., [49,83-86]). This would possibly further reduce the amount of times, when shared control is necessary for intervention. However, already in our experimental setting, participants rarely needed shared control sensors and most of them had full control on the user side.

## Conclusion

We explored tactile ERP-BCI based online wheelchair control in a virtual environment. Participants overall gained high accuracy levels in copy tasks and when navigating through the virtual environment. Importantly, 11 participants finished the requested task, i.e. successfully navigated along four checkpoints. Most participants did not require shared control sensors. In conclusion, our results prove tactile ERP-BCIs feasible for wheelchair control. Yet we discovered and discussed a number of issues to be addressed and solved in future research. Most importantly, data have to be collected with the targeted patient group in the iterative process of user-centered BCI development.

## Competing interests

The authors declare that they have no competing interests.

## Authors’ contributions

TK and AK designed the study. TK and AH programmed and tested the setup. AH collected the data. TK and AH analyzed the data. TK, AH and AK discussed the results. TK drafted the manuscript. AH and AK revised the manuscript. All gave their approval to the final version to be published.
